# Anxiety and Depression Scales in Patients with Fibromyalgia: Correlation with Disease Symptomatology

**DOI:** 10.3390/jcm14165867

**Published:** 2025-08-20

**Authors:** Javier Amezqueta, Javier Nicolás García González

**Affiliations:** 1 Department of Medicine, University of Navarra, 31009 Pamplona, Spain; 2 Department of Internal Medicine, Clinica Universidad de Navarra, University of Navarra, 31009 Pamplona, Spain

**Keywords:** fibromyalgia, anxiety, depression

## Abstract

**Background/Objectives:** Fibromyalgia (FM) is a chronic condition characterized by widespread pain and a broad range of associated symptoms. Among these, significant psychiatric comorbidities—particularly anxiety and depression—are frequently observed. This study aimed to evaluate the relationship between the severity of fibromyalgia and symptoms of anxiety and depression. We correlated the scores from the Hospital Anxiety and Depression Scale (HADS) with those from various fibromyalgia symptom questionnaires, including the Widespread Pain Index (WPI), the Symptom Severity Score (SS), and the 12-Item Short Form Survey (SF-12). **Methods:** A sample of 59 FM patients treated at the Department of Internal Medicine at Clínica Universidad de Navarra was assessed using HADS, SS, SF-12, and WPI scales. Spearman correlation analysis was performed to examine the relationships among the scale scores. **Results:** The analysis revealed a statistically significant moderate correlation (r = 0.442; *p* < 0.01) between HADS and the Symptom Severity Score, a statistically significant strong negative correlation (r = −0.678; *p* < 0.01) between HADS and the Mental Component Summary (MCS-12), and a statistically significant moderate negative correlation (r = −0.417; *p* < 0.01) between the SS Score and the Physical Component Summary (PCS-12). **Conclusions:** These findings suggest a clear association between anxiety and depressive symptoms, as measured by HADS, and the severity of fibromyalgia.

## 1. Introduction

Fibromyalgia (FM) is a disease characterized by chronic widespread pain of unknown cause, associated with significant psychiatric comorbidity [1,2]. The understanding of this condition has evolved over the years, leading to the addition of various signs and symptoms beyond pain, which have helped define it as a syndrome. FM has a multifactorial origin, including genetic, neurogenic, immunogenic, and psychogenic mechanisms [3]. The development of FM is influenced by genetic factors, as evidenced by familial aggregation, a 50% concordance rate in twins, and the presence of polymorphisms associated with the disease, such as the serotonin transporter and the dopamine D4 and 5 HT2A receptors.

Fibromyalgia (FM) is occasionally observed in conjunction with other conditions that may function as triggering factors, including osteoarthritis, other rheumatologic disorders, physical trauma, exercise, and hormonal imbalances such as thyroid dysfunction (T4).

Moreover, a potential temporal association between psychological stressors and fibromyalgia (FM) has been proposed, which may contribute to an increased risk of developing the syndrome concurrently.

Several authors have proposed that FM constitutes a “central pain disorder”, arising from a dysfunction in the central nervous system (CNS) that disrupts the psysiological pain modulation mechanisms [4]. This dysregulation manifests as heightened pain sensitivity, likely resulting from elevated concentrations of nociceptive agents (e.g., glutamate, aspartate, and substance P) and reduced levels of antinociceptive neurotransmitters such as serotonin and norepinephrine.

In addition to this central sensitization, other mechanisms have been implicated in the chronicity of FM, including somatomedin C deficiency and disturbances in hypothalamic and autonomic nervous system function. These physiological abnormalities are associated with a broad constellation of symptoms, notably fatigue; cognitive impairmen; non-restorative sleep; and psychiatric comorbidities such as anxiety, depression, and stress—all frequently reported by FM patients. In addition, the relationship between simple chronic pain and chronic pain in multiple locations and dementia has been assessed in many studies conducted to date.

Although pain is the cardinal symptom of FM, it is characteristically accompanied by a range of other manifestations, including persistent fatigue, sleep disturbances, cognitive dysfunction, and psychological distress [5]. Fatigue is particularly debilitating; it often begins upon awakening and intensifies with minimal physical or mental exertion. Many patients experience exacerbations termed “exhaustion crises”, which can be so severe they result in temporary functional incapacitation. Even outside of these episodes, fatigue significantly impairs daily functioning.

Sleep architecture in FM patients is typically non-restorative and frequently disrupted by nocturnal awakenings, periodic limb movements (myoclonus), and occasionally akathisia. Less commonly, patients report neurological symptoms (e.g., parestesia and disequilibrium), neurocognitive deficits (e.g., impaired concentration and reduced short-term memory), and autonomic dysfunction (e.g., arrhythmias and vagally mediated hypotension).

Given the absence of specific biological markers, FM diagnosis is reliant on clinical criteria. To improve diagnostic precision and facilitate symptom quantification, various assessment instruments have been developed. Among the most widely used are the Widespread Pain Index (WPI) and the Symptom Severity (SS) Score, which are integral to current diagnostic frameworks.

In 2010, formal diagnostic criteria for FM were introduced, requiring that patients satisfy the following conditions:

1. A WPI score ≥ 7 combined with an SS score ≥ 5, or alternatively, a WPI between 3 and 6 with an SS score ≥ 9.
2. Persistence of symptoms for a minimum duration of three months, with relatively stable intensity over time.
3. The pain experienced must not be better explained by another underlying medical condition [6].

Management of fibromyalgia is multifaceted, centering on three key objectives: pain control, sleep restoration, and psychological stabilization. Contemporary clinical guidelines advocate for an integrative approach, incorporating pharmacological therapy alongside psychoeducation, psychiatric evaluation, and tailored physical exercise regimens [7]. However, no universally accepted treatment algorithm currently exists; therapeutic strategies must be individualized based on symptomatology and disease trajectory [8].

Only three agents have received approval from the U.S. Food and Drug Administration (FDA) for the treatment of FM: the gabapentinoid pregabalin, and the serotonin-norepinephrine reuptake inhibitors (SNRIs) milnacipran and duloxetine [9]. Notably, none of these agents are approved for FM management by the European Medicines Agency (EMA), which recommends non-pharmacological interventions as the first-line treatment modality, in alignment with the guidelines set forth by the European League Against Rheumatism (EULAR) [10].

Among non-pharmacological therapies, the most effective evidence-based interventions include aerobic exercise, cognitive behavioral therapy (CBT), patient education, and interdisciplinary care models.

The relationship between FM and psychiatric comorbidities—particularly anxiety and depression—is well documented, with these conditions constituting hallmark features within the broader clinical spectrum of Chronic Fatigue Syndrome. Epidemiological data reveal significantly elevated rates of depression (20–80%) and anxiety (13–63.8%) in FM patients compared to the general population [8]. These psychiatric symptoms may exacerbate the severity of pain and other manifestations, thereby compounding the disease burden and negatively impacting overall patient outcomes [6].

Neurobiological studies have identified complex interactions between nociceptive regulatory systems and emotional processing pathways, suggesting that dysregulation in these systems impairs pain modulation and may amplify pain perception. In this context, anxiety emerges as both a precipitating and perpetuating factor in FM, with higher anxiety levels correlating with increased pain intensity [8].

Similarly, depression is widely recognized as a contributor to pain chronicity [8]. Furthermore, as noted earlier, some psychological stressors appear temporally associated with the onset and exacerbation of FM symptoms.

In conclusion, fibromyalgia represents a profound medical and societal challenge due to its high prevalence, elusive etiology, broad symptom spectrum, and complex management requirements. This has spurred considerable interest in elucidating its patho-physiology to inform more effective treatment paradigms. Although substantial literature highlights the interplay between FM and affective disorders, there remains a dearth of research directly examining the relationship between psychiatric symptom severity and the intensity of FM manifestations.

This study aims to address this gap by evaluating whether the severity of anxiety and depression symptoms in patients with FM correlates with the clinical severity of the syndrome, as measured by standardized diagnostic instruments.

## 2. Materials and Methods

This study was conducted on a cohort of 59 patients diagnosed with fibromyalgia (FM) who received care at the Department of Internal Medicine, Clínica Universidad de Navarra, between 2018 and 2023. Patients were diagnosed using the American College of Rheumatology (ACR) criteria.

Clinical information was collected from each participant using standardized and validated instruments, including the Widespread Pain Index (WPI), the Symptom Severity Score (SS score), and the Short Form-12 Health Survey (SF-12). Additionally, the Hospital Anxiety and Depression Scale (HADS) was administered during the same clinical visit. Each questionnaire was completed only once per participant, and the responses were used to construct an anonymized research database.

The Widespread Pain Index (WPI), a key diagnostic tool in FM, records patient-reported pain or muscle fatigue in 19 anatomical regions over the preceding seven days. Each region contributes one point, yielding a total score ranging from 0 to 19. Although this method is subjective and the reported information cannot always be objectively verified, WPI remains essential in any study involving FM due to its consistent application in both clinical and research settings.

The Symptom Severity Score (SS score) complements WPI by assessing the severity and range of symptoms associated with FM. It consists of two main components:

- The first section evaluates three primary symptoms over the past week: fatigue, cognitive symptoms, and non-restorative sleep. Each item is rated on a four-point Likert scale: 0 (no problem), 1 (mild/intermittent), 2 (moderate), and 3 (severe). The total score in this section ranges from 0 to 9 points.
- The second section assesses the presence of additional symptoms experienced during the same timeframe. A list of 40 common FM-related symptoms is presented, and scores are assigned as follows: 0 (no symptoms), 1 (1–10 symptoms), 2 (11–24 symptoms), and 3 (25 or more symptoms).

The total SS score thus ranges from 0 to 12 points, providing a standardized measurement of overall symptom burden in FM patients.

The SF-12 Health Survey, unlike the WPI and SS score, evaluates general health-related quality of life, encompassing physical, mental, and social functioning. It is widely used in epidemiological and clinical research to facilitate comparisons among diverse patient populations. Given the chronic and multifactorial nature of FM, SF-12 was selected to assess the overall health impact of the disease. For analytical purposes, SF-12 was divided into two components—the Physical Component Summary (PCS-12) and the Mental Component Summary (MCS-12)—enabling examination of their correlations with the other clinical and psychological variables studied.

The Hospital Anxiety and Depression Scale (HADS) was included to screen for anxiety and depression symptoms in patients without previously diagnosed psychiatric disorders. This self-report instrument contains 14 items, evenly divided into two subscales: anxiety (HADS-A) and depression (HADS-D). Each item is rated from 0 to 3, with each subscale yielding a total score between 0 and 21. A score of 8 or higher suggests clinically significant symptoms, while a score of 11 or higher indicates moderate-to-severe psychiatric distress. HADS was selected due to its widespread use, validated psychometric properties, and relevance in FM populations where psychological comorbidity is common and clinically meaningful.

All collected data were entered and processed using IBM SPSS Statistics, Version 28. Variables were properly formatted by defining the decimal precision, column width, variable type, and coding for missing values.

The statistical analysis consisted of the following steps:

- Normality testing, to determine the distribution of the dataset.
- Descriptive statistics, including means, minimum and maximum values, and standard deviations for each variable.
- Inferential statistical analyses, to assess the correlations between clinical, psychological, and health status variables, in accordance with the primary hypothesis of the study.

## 3. Results

From the outset, due to the nature of the variables used (scales), it was anticipated that the sample would not follow a normal distribution. However, normality tests were conducted to objectively characterize the sample and determine the appropriate type of correlation analysis. By examining the significance level of the Shapiro-Wilk test, it was confirmed that the sample does not follow a normal distribution. Secondly, a descriptive statistical analysis of the sample was conducted. This analysis included missing values, which amounted to a total of three patients, as they were the only ones who did not complete the Health Index. Consequently, two variables—PCS-12 and MCS-12—had a reduced sample size (Table 1, Table 2 and Table 3).

Thirdly, a Spearman correlation analysis was performed due to the previously mentioned characteristics of the sample. For this test, *p*-values lower than 0.05 were considered statistically significant. Regarding the correlation coefficients, the following classification was applied: values above 0 indicate a positive correlation, whereas values below 0 indicate a negative correlation. In terms of absolute values, correlations were categorized as follows: 0–0.10 indicated no correlation, 0.10–0.29 indicated a weak correlation, 0.30–0.50 indicated a moderate correlation, and 0.50–1.00 indicated a strong correlation.

## 4. Discussion

This study was conducted with the objective of exploring potential correlations between the various clinical manifestations of fibromyalgia, as assessed by the scores from different questionnaires completed by these patients, with a particular focus on the correlation between disease severity and anxious and depressive symptomatology. The Results section presents the findings of the statistical analysis performed.

Firstly, a moderate and statistically significant correlation was identified between two of the diagnostic questionnaires used for fibromyalgia, the Widespread Pain Index (WPI) and the Symptom Severity Score (SS Score). This correlation is logical given the content of these diagnostic tools, as both collect information related to symptoms of the disease.

Regarding the correlations calculated between both the WPI and SS score with the HADS scale, two different scenarios were observed. Firstly, the correlation between WPI and HADS was weak, with a tendency toward statistical significance. One possible explanation for the lack of a statistically significant correlation could be the small sample size used in this study. Another possible reason could be the presence of confounding factors such as socioeconomic status, education level, employment status, or even sex, all of which have been shown to influence the studied hypothesis [11].

Secondly, a moderate-to-strong, statistically significant correlation was found between the SS score and HADS scale scores. These findings align with the existing literature, which has demonstrated that fibromyalgia patients with severe anxiety and depression tend to report higher pain perception and more severe symptoms compared to those with mild or moderate levels of anxiety and depression.

One of the most influential studies to date, aimed at confirming this correlation, utilized a set of questionnaires different from those used in our study but reached the same conclusion: fibromyalgia symptoms, assessed in that case using the Fibromyalgia Impact Questionnaire (FIQ) score, correlate with symptoms of anxiety and depression, which were evaluated in that study using the Beck Depression Inventory (BDI) and the Beck Anxiety Inventory (BAI) [12].

Thirdly, regarding the two variables from our study that make up the SF-12 Score—namely, the Physical Component Summary (PCS-12) and the Mental Component Summary (MCS-12)—several important correlations were identified. Both variables showed a moderate, statistically significant negative correlation with the SS score. This correlation aligns with clinical evidence, indicating that as the severity of fibromyalgia increases, there is a significant deterioration in patients’ perceived overall health status, both physically and mentally.

Fourthly, regarding the correlation between the HADS scale and the two components derived from the SF-12 score, two very different results were obtained. No correlation was found between HADS and PCS-12. However, a strong negative correlation was observed between HADS and MCS-12. This finding is consistent with the existing literature.

In the most recent study analyzing this relationship, a sample of 120 patients was used to assess the correlation between SF-12 and the MOODS-SR Lifetime Questionnaire. A significant correlation was found between the Health Index (SF-12) and one of the MOODS-SR Lifetime Questionnaire subscales, specifically MOODS-Depressive. Although the tool used to assess anxiety and depression symptoms differed (MOODS-SR instead of the HADS scale) and the correlation was calculated based on the total SF-12 rather than its components (PCS-12 and MCS-12), the association between the two variables produced highly comparable results.

Moreover, despite differences in sample size, the mean scores for these two variables were: 29.45 for PCS-12 and 37.54 for MCS-12 [13], values very similar to those obtained in our study (PCS-12: 26.5 and MCS-12: 37.54). These findings suggest that the Mental Component Summary (MCS-12) has a greater impact than the Physical Component Summary (PCS-12) in the observed correlation between the HADS scale and SF-12 [13].

As this is a cross-sectional study, only correlations and means could be calculated, making the temporal sequence one of its main limitations. Because data were collected during a single visit, it is impossible to determine whether patients exhibited severe symptoms before the onset of anxiety–depressive symptoms. This means that, within the calculated correlations, no causal relationship can be established between the severity of anxious and depressive symptoms and the progression of fibromyalgia, or vice versa. Future prospective studies would be beneficial to closely track the evolution of the disease and assess how anxiety and depression influence its course.

Additionally, no clinical data were collected on the study participants. That is, information regarding gender, treatments (both for FM symptoms and other comorbidities), the presence of associated pathologies, age, or disease duration is unknown. These variables could have influenced the analysis results.

Although data loss was minimal, some patients left certain questionnaire items unanswered. The approach taken for patients who completed more than half of the test was to calculate the average score for the missing responses, following established protocols. However, three cases did not complete SF-12, making it the only variable with a reduced sample size.

## 5. Conclusions

1. A moderate-to-strong and statistically significant correlation was found between the Symptom Severity Score (SS score) and the Hospital Anxiety and Depression Scale (HADS). This finding suggests that higher levels of anxiety and depressive symptoms are associated with a greater severity of fibromyalgia symptoms.
2. As mentioned in the introduction to this article, there are still unknowns surrounding the possible genetic, endocrine, and biological components that may be involved in the association between FM and psychiatric disorders.
3. A weak-to-moderate correlation with a trend toward significance was observed between the Widespread Pain Index (WPI) and HADS scale.
4. Although a causal relationship between anxiety, depression, and fibromyalgia severity cannot be determined, the strong association demonstrated between these symptoms underscores the need for further research. Conducting prospective studies with a larger sample size could help clarify the role of anxiety and depressive symptoms in the progression and severity of fibromyalgia. The importance of the latter is due to the fact that it could influence the therapeutic approach to be followed in patients with this pathology.

## 6. Patents

There are no patents resulting from the work reported in this manuscript.

## Figures and Tables

**Table 1:** Normality Test.

	Kolgomorov-Smirnov			Shapiro-Wilk		
	Estadistic	gl	Sig.	Estadistic	gl	Sig.
WPI	0.110	56	0.087	0.979	56	0.415
SS1	0.238	56	<0.001	0.915	56	<0.001
SS2	0.401	56	<0.001	0.679	56	<0.001
SS12	0.224	56	<0.001	0.915	56	<0.001
PCS12	0.066	56	0.200 *	0.982	56	0.557
MCS12	0.159	56	0.001	0.958	56	0.047
HADSA	0.083	56	0.200 *	0.980	56	0.458
HADSD	0.098	56	0.200 *	0.982	56	0.568
HADS	0.089	56	0.200 *	0.984	56	0.681

* Lower bound of the true significance.

**Table 2:** Descriptive statistic Study.

	N	Minimum	Maximum	Mean	Standard Desviation
WPI	59	3	19	11.58	3.611
SS1	59	2	9	6.08	1.976
SS2	59	1	3	1.88	0.0494
SS12	59	3	11	7.97	2.189
PCS12	56	12.49956	48.56097	26.4992729	7.98552349
MCS12	56	16.33011	66.05597	37.5441816	12.14702754
HADSA	59	1	20	10.34	4.558
HADSD	59	2	19	9.61	3.900
HADS	59	4	39	19.95	7.523
VALID N	56				

**Table 3:** Spearman correlation.

Correlación de Spearman	Valores	WPI	SS1	SS2	SS12	PCS12	MCS12	HADSA	HADSD	HADS
WPI	Coef. Corre. ^1^	1	0.288 *	0.430 **	0.375 **	−0.144	−0.297 *	0.191	0.239	0.248
	*p* ^2^		0.027	<0.001	0.003	0.402	0.026	0.148	0.069	0.058
	N ^3^	59	59	59	59	56	56	59	59	59
SS1	Coef. Corre.	0.288 *	1	0.303 *	0.965 **	−0.402 **	−0.287 *	0.251	0.469 **	0.383 **
	*p*	0.027		0.020	<0.001	0.002	0.032	0.056	<0.001	0.003
	N	59	59	59	59	56	56	59	59	59
SS2	Coef. Corre.	0.430 **	0.303 *	1	0.522 **	−0.184	−0.309 *	0.324 *	0.389 **	0.389 **
	*p*	<0.001	0.020		<0.001	0.174	0.021	0.012	0.002	0.002
	N	59	59	59	59	56	56	59	59	59
SS12	Coef. Corre.	0.375 **	0.965 **	0.522 **	1	−0.417 **	−0.329 *	0.300 *	0.528 **	0.442 **
	*p*	0.003	<0.001	<0.001		0.001	0.013	0.021	<0.001	<0.001
	N	59	59	59	59	56	56	59	59	59
PCS12	Coef. Corre.	−0.144	−0.402 **	−0.184	−0.417 **	1	−0.281 *	0.216	−0.144	0.055
	*p*	0.402	0.002	0.174	0.001		0.036	0.110	0.289	0.685
	N	56	56	56	56	56	56	56	56	56
MCS12	Coef. Corre.	−0.297 *	−0.287 *	−0.309 *	−0.329 *	−0.281 *	1	−0.566 **	−0.643 **	−0.678 **
	*p*	0.026	0.032	0.021	0.013	0.036		<0.001	<0.001	<0.001
	N	56	56	56	56	56	56	56	56	56
HADSA	Coef. Corre.	0.191	0.251	0.324 *	0.300 *	0.216	−0.566	1	0.561 **	0.912 **
	*p*	0.148	0.056	0.012	0.021	0.110	<0.001		<0.001	<0.001
	N	59	59	59	59	56	56	59	69	59
HADSD	Coef. Corre.	0.239	0.469 **	0.389 **	0.528 **	−0.144	−0.643 **	0.561 **	1	0.840 **
	*p*	0.069	<0.001	0.002	<0.001	0.289	<0.001	<0.001		<0.001
	N	59	59	59	59	56	56	59	59	59
HADS	Coef. Corre.	0.248	0.383 **	0.389 **	0.442 **	0.055	−0.678 **	0.912 **	0.840 **	1
	*p*	0.058	0.003	0.002	<0.001	0.685	<0.001	<0.001	<0.001	
	N	59	59	59	59	56	56	59	59	59

**Table 3 Spearman correlation foundthrough statistical analysis. ^1^ Coef Corre:** Correlation coefficient, **^2^ *p*:** Two-tailed significance value (*p*-value), **^3^ N:** Sample size * The correlation is significant at the 0.05 level (two-tailed). ** The correlation is significant at the 0.01 level (two-tailed).

## Data Availability

The original contributions presented in this study are included in the article. Further inquiries can be directed to the corresponding author.
